# Glycerol: An Optimal Hydrogen Source for Microwave-Promoted Cu-Catalyzed Transfer Hydrogenation of Nitrobenzene to Aniline

**DOI:** 10.3389/fchem.2020.00034

**Published:** 2020-01-29

**Authors:** Maria Jesus Moran, Katia Martina, Georgios D. Stefanidis, Jeroen Jordens, Tom Van Gerven, Vincent Goovaerts, Maela Manzoli, Carlo Groffils, Giancarlo Cravotto

**Affiliations:** ^1^Dipartimento di Scienza e Tecnologia del Farmaco, University of Turin, Turin, Italy; ^2^Department of Chemical Engineering KU Leuven, Leuven, Belgium; ^3^MEAM Microwave Test Center, Herk-de-Stad, Belgium

**Keywords:** glycerol, microwaves, ultrasound, transfer hydrogenation, copper nanoparticles

## Abstract

The search for sustainable alternatives for use in chemical synthesis and catalysis has found an ally in non-conventional energy sources and widely available green solvents. The use of glycerol, an abundant natural solvent, as an excellent “sacrificial” hydrogen source for the copper-catalyzed microwave (MW)-promoted transfer hydrogenation of nitrobenzene to aniline has been investigated in this work. Copper nanoparticles (CuNPs) were prepared in glycerol and the efficacy of the glycerol layer in mediating the interaction between the metal active sites has been examined using HRTEM analyses. Its high polarity, low vapor pressure, long relaxation time, and high acoustic impedance mean that excellent results were also obtained when the reaction media was subjected to ultrasound (US) and MW irradiation. US has been shown to play an important role in the process via its ability to enhance CuNPs dispersion, favor mechanical depassivation and increase catalytic active surface area, while MW irradiation shortened the reaction time from some hours to a few minutes. These synergistic combinations promoted the exhaustive reduction of nitrobenzene to aniline and facilitated the scale-up of the protocol for its optimized use in industrial MW reactors.

## Introduction

The development of more direct catalytic approaches to the synthesis of chemical products is a key goal in achieving chemical sustainability. A synthetic process that combines heterogeneous catalysis in a green sustainable medium and non-conventional selective MW heating to promote fast chemical transformations is an appealing approach to the development of environmentally benign organic transformations. MW processing offers many advantages compared to classic conductive heating because the intrinsic properties of heat transfer by volumetric dielectric heating (Cravotto and Cintas, 2017). Fast heating rates, short processing times, instantaneous and precise electronic control, and clean heating profile are the main features of MW promoted processes (Rattanadecho and Makul, 2016). Since late 1960s the development of industrial applications of MW heating was mainly for drying and other thermal treatments. Today MW technology is exploited in several areas: drying (Feng et al., 2012), calcination, decomposition, polymerization (Kempe et al., 2011), chemical process control (Bhusnure et al., 2015), and production of nanomaterials (Dabrowska et al., 2018). In spite of the outstanding achievements in MW-assisted organic synthesis, its industrialization is limited to few applications. The exploitation of MW toward a sustainable economy, the discovery of novel waste-to-product approaches and the development of large scale protocol, that can provide products on a kg scale is the way to bypass the negative impact of the relatively high operating cost of MW processes, paving the way for a fully accepted technology.

One way to pursue green synthesis is to improve the sustainable nature of the solvents, as they are directly responsible for the major environmental drawback generated by chemical processes. The use of bio-based and eco-friendly alternative solvents (Gandeepan et al., 2019) has been developed and evaluated over recent decades. Glycerol (1,2,3-propanetriol) is a common natural solvent that is rich in functionalities and is obtained in very large amounts as a co-product in biodiesel production (Cintas et al., 2014; Sudhakar et al., 2016). The rapid development of the biodiesel industry has resulted in an increase in glycerol production yields and a supply of low cost technical grade glycerol with a final purity of around 80–95% (Zhou et al., 2008). More than 90% of the glycerol used today is refined to give purities of higher than 97%, and the process can take purity up to from 99.5 to 99.7%. Intensive research is conducted to find ways to valorise glycerol and many fields of interest focus on its transformation into chemicals and hydrocarbon fuels (Dodekatos et al., 2018). Furthermore, its use as a convenient green reaction medium has been widely documented (Wolfson et al., 2009; Díaz-Álvarez and Cadierno, 2013; Díaz-Álvarez et al., 2014; Tagliapietra et al., 2015; Santoro et al., 2017).

Reductive transformations are a vast class of chemical reactions that can be achieved both by catalytic processes that utilize molecular hydrogen, and others that use a less reactive hydrogen source (Filonenko et al., 2018). Rather than pressurized hydrogen, it is metal-catalyzed dehydrogenative transformations that are paving the way for sustainable processes with a high degree of control over selectivity and reaction rate. Coupled transfer hydrogenation–dehydrogenation reactions involve the transfer of one hydrogen from a donor molecule (alcohols, ethers and amines) to an unsaturated bond. A wide range of hydrogen-transfer reactions have been studied thus far (Baráth, 2018), with primary and secondary alcohols usually being preferred for use. There is some preference for secondary alcohols as they are better donor molecules than primary alcohols because of the sigma inductive electronic effect. Glycerol can also be successfully employed as an environmentally benign “donor solvent” in transfer hydrogenation–dehydrogenation reactions for the reduction of ketones, aldehydes, olefins and aromatic nitro compounds. The preferred metallic catalytic systems for these processes are based on Ru, Pd, Ir, Ni, and bimetallic catalysts (Wolfson et al., 2009; Díaz-Álvarez and Cadierno, 2013).

The reduction of aromatic nitro compounds is an important transformation that has been widely studied because anilines are important building blocks in the synthesis of pharmaceuticals and agrochemicals. Selective and complete reductions of nitrobenzenes in the presence of glycerol, used as a “sacrificial” hydrogen source, have been performed with Ni Raney (Wolfson et al., 2009) and in the presence of a recyclable catalyst made of magnetic ferrite-Nickel NPs (Gawande et al., 2012). Furthermore, bio-based glycerol has been exploited in the Ru-catalyzed, one-pot synthesis of imine and amine using nitrobenzene and alcohol as the starting materials (Cui et al., 2012, 2013). An example of light-driven nitrobenzene reduction to aniline by transfer hydrogenation of glycerol has been described catalyzed by Pd/TiO_2_ (Zhou et al., 2015). Glycerol has been also utilized to prepare 1,2,3-trimethoxypropane a green alternative for petroleum-based solvents, such as THF, toluene and dichloromethane. 1,2,3-Trimethoxypropane has given good results in the Fe(acac)_3_-catalyzed transfer hydrogenation of carboxylic acids, nitriles, esters and nitrobenzene (Sutter et al., 2013).

Despite its main disadvantage, i.e., its high viscosity at room temperature, glycerol is an optimal solvent for catalysis purposes because of its high polarity and capacity to remain in the liquid phase over a large temperature range (from 17.8 to 290°C). Moreover, it has low vapor pressure, a long relaxation time and high acoustic impedance, meaning that it can be used under MW and US irradiation conditions. In fact, glycerol has a high loss factor, or loss tangent (tan δ = 0.651), at the standard MW frequency (2.45 GHz), which is indicative of high MW absorption and rapid heating. Several successful examples of MW-promoted organic syntheses in glycerol have therefore been described in the literature (Cravotto et al., 2011; Cintas et al., 2014). Glycerol can be used under sonochemical conditions, although greater amounts of energy must be supplied to overcome the cohesive forces in the liquid, as it is a viscous solvent. Similarly to other polyols (e.g., ethylene glycol and polyethylene glycol), glycerol can act as both a solvent and reducing agent of metal precursors, and several applications have been developed in the field of metal-nanoparticle synthesis. Furthermore, glycerol can act as a stabilizer of nanometric species, leading to the straightforward recycling of the catalytic phase (Chahdoura et al., 2014). The conventional and MW-assisted preparation of NPs in glycerol has already been described in the literature and this field of interest is continuously growing (Wang et al., 2018; Ghosh et al., 2019; Parveen et al., 2019; Vinodhini et al., 2019). CuNPs are an efficient source of Cu, but their potential applicability is restricted by copper's inherent instability under atmospheric conditions (Gawande et al., 2016). Cu(0)NPs have been efficiently prepared in glycerol (Dang-Bao et al., 2017), and some polyol-stabilized CuNPs have been used in the reduction of nitrobenzene (Saha and Ranu, 2008; Duan et al., 2012; Santhanalakshmi and Parimala, 2012). Although several applications for copper catalysis in transfer hydrogenation have already been reported in the literature (Štefane and Požgan, 2014; Fan et al., 2018; Zhang and Li, 2019), the use of CuNPs for nitrobenzene reduction via transfer hydrogenation has not received much attention, to the best of our knowledge (Feng et al., 2014). In fact, the common approach to nitro benzene reduction by Cu catalysis is performed in the presence of NaBH_4_, which is used as a hydride donor (Wu et al., 2013; Aditya et al., 2017; de Souza et al., 2017).

Herein, glycerol has been exploited as an efficient capping agent in the production of CuNPs, and as a solvent and hydrogen donor in the Cu-catalyzed reduction of nitrobenzene derivatives. Non-conventional, non-contact energy sources have been utilized to create new transfer hydrogenation processes that benefit from additional actuation via intensified mass and heat transfer. The ability of dielectric heating and US irradiation to maximize catalyst dispersion have been explored with the aim of enhancing reaction rate. All of the activities that are presented herein have the final aim of developing a knowledge-based strategy and selecting appropriate technologies for the scale up of the optimized reaction to an industrial MW instrument.

## Results and Discussion

The high reactivity of CuNPs and their recyclability have driven us to optimize the nitrobenzene reduction to aniline in the presence of CuNPs. NPs were prepared using a slight modification to a published procedure (Zhang et al., 2009). We decided to avoid the use of poly *N*-vinyl pyrrolidone (PVP) when investigating the efficacy of glycerol alone as a means to cap and stabilize the NPs. Cu(0) NPs were prepared according to the “bottom-up” approach; by dispersing CuSO_4_ in a basic solution (pH 11) of water and glycerol (5:1), using the polyol as the stabilizer and solvent. NaBH_4_ was immediately added and the deep blue solution became a colorless one in which the dark NPs could be identified (Supplementary Figure 7). Transmission electron microscopy (TEM) and particle-size distribution analyses were performed to characterize the prepared catalyst.

**Figure 1:**
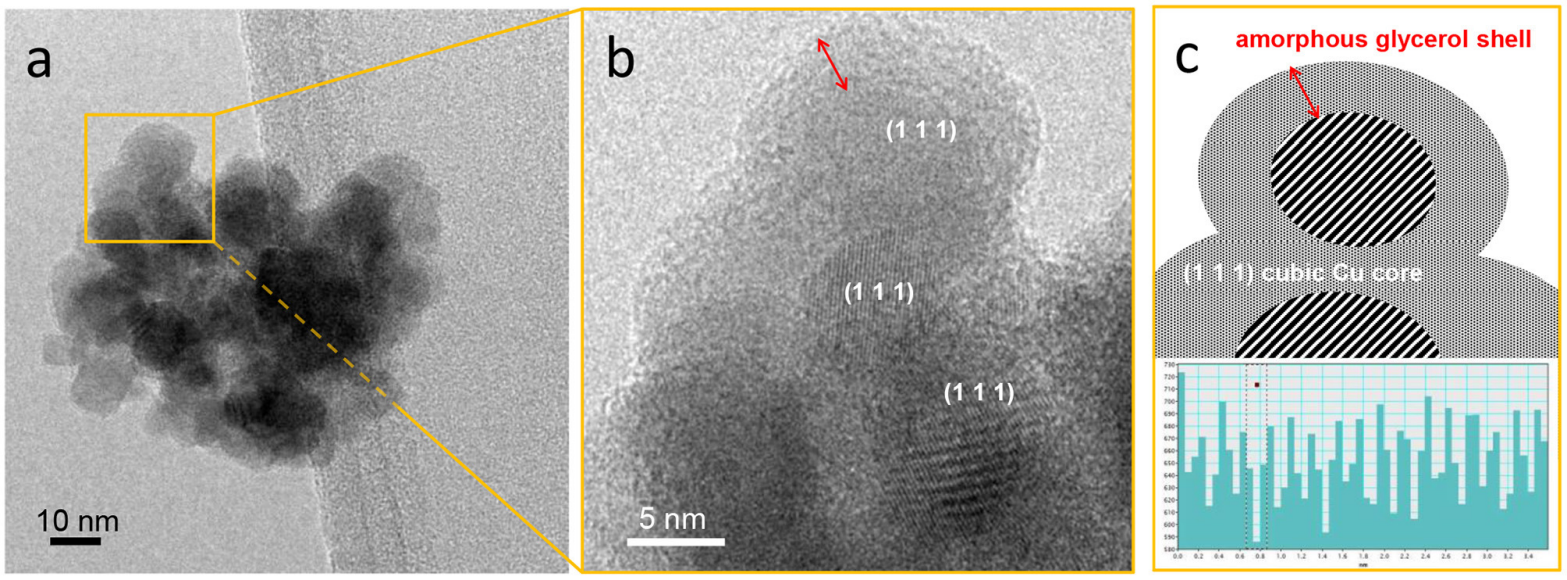
**(a)** HRTEM image of CuNPs, **(b)** zoom-in of the region shown in **(a)**, and **(c)** schematic representation of nanoparticle morphology and graph reporting the measurements of the spacings between the observed diffraction fringes. Instrumental magnification 3,00,000 ×.

CuNPs with a roundish shape and an average size of 10.2 ± 3.0 nm were obtained. Moreover, the NPs tended to form aggregates (Figure 1a). HRTEM analyses showed that metallic crystalline Cu is formed, as demonstrated by the presence of diffraction fringes with spacing associated to the (1 1 1) plane of metallic Cu in the cubic phase (JCPDS file number 00-001-1242), as reported in Figures 1b,c. Moreover, an amorphous layer was observed around the NPs (Figure 1b, red arrow). The characteristics of the synthetic procedure and the contrast phase make it reasonable to propose that the CuNPs are coated with a glycerol layer that acts both as a protecting agent and stabilizer. Indeed, the NPs did not coalesce under the electronic beam of the instrument, and presumably do not do so under the reaction conditions, proving that they are quite stable. The synthesized NPs can be described as having a core-shell morphology, in which the core is made up of crystalline Cu^0^ and the shell by glycerol molecules, as depicted in Figure 1c.

The existence of this morphology suggests that the glycerol layer mediates interactions between the metal active sites and the reagents. Moreover, besides having a stabilizing function, the glycerol layer can moderately promote NP dispersion in the glycerol solvent, therefore enhancing the active metallic surface area of the catalyst.

**Scheme 1:**
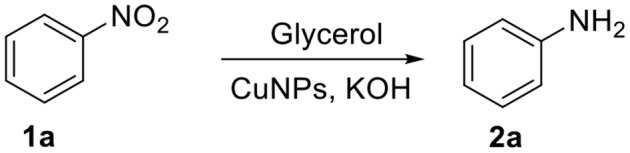
CuNPs-catalyzed reduction of nitrobenzene (**1a**) using glycerol as reducing agent to obtain aniline (**2a**).

The reduction of nitrobenzene (1a) to aniline (2a) was performed in the presence of CuNPs and glycerol was used as the solvent and “sacrificial” hydrogen source (Scheme 1).

As shown in Table 1, the reaction parameters were optimized by varying the nature of the base, the reaction temperatures and catalyst amounts. Several bases were tested: KOH, NaOH, K_2_CO_3_, CsCO_3_, and all the reactions were performed at 130°C, taken as the optimal reaction temperature, while the reaction time was fixed at 5 h. KOH showed the best results (Table 1, entry 5). Excellent results were obtained in the presence of 2 eq of base and 5 mol% of CuNPs.

**Table 1:** Optimization of the Cu-Catalyzed nitrobenzene reduction in glycerol.

**Entry**	**Reaction conditions**	**Temperature and time**	**Yields^a^**
1	K_2_CO_3_ (2 eq), CuNPs (5 mol%)	130°C, 5 h	80
2	CsCO_3_ (2 eq), CuNPs (5 mol%)	130°C, 5 h	2
3	-, CuNPs (5 mol%)	130°C, 5 h	1
4	NaOH (2 eq), CuNPs (5 mol%)	130°C, 5 h	87
5	KOH (2 eq), CuNPs (5 mol%)	130°C, 5 h	99
6	KOH (1 eq), CuNPs (5 mol%)	130°C, 5 h	62
7	KOH (2 eq), CuNPs (2 mol%)	130°C, 5 h	67
8	KOH (2 eq), CuNPs (10 mol%)	130°C, 5 h	98
9	KOH (2 eq), CuNPs (5 mol%)	80°C, 15 h	20
10	KOH (2 eq), CuNPs (5 mol%)	50°C, 15 h	2
11	KOH (2 eq), CuNPs (5 mol%)	130°C, 2 h	99
12^b^	KOH (2 eq), CuNPs (5 mol%)	US, 1 h	99
13^c^	KOH (2 eq), CuNPs (5 mol%)	MW 130°C, 30 min	99

*Reaction conditions: nitrobenzene (1 mmol), base (2 mmol), glycerol (40 mmol), CuNPs (5 mol%), oil bath, 5h*.

a *Determined by GC-MS*.

b *UP50Hz, F(kHz):30, P(W):50*.

c *CEM Mars 5, 130°C Pmax = 400 W*.

The influence of US irradiation, given that the CuNPs usually aggregate, was investigated in order to improve reaction rate. US is known for its capacity to enhance particle dispersion and favor mechanical depassivation (Banerjee, 2019). Particle-size distribution was therefore measured before and after US treatment.

**Figure 2:**
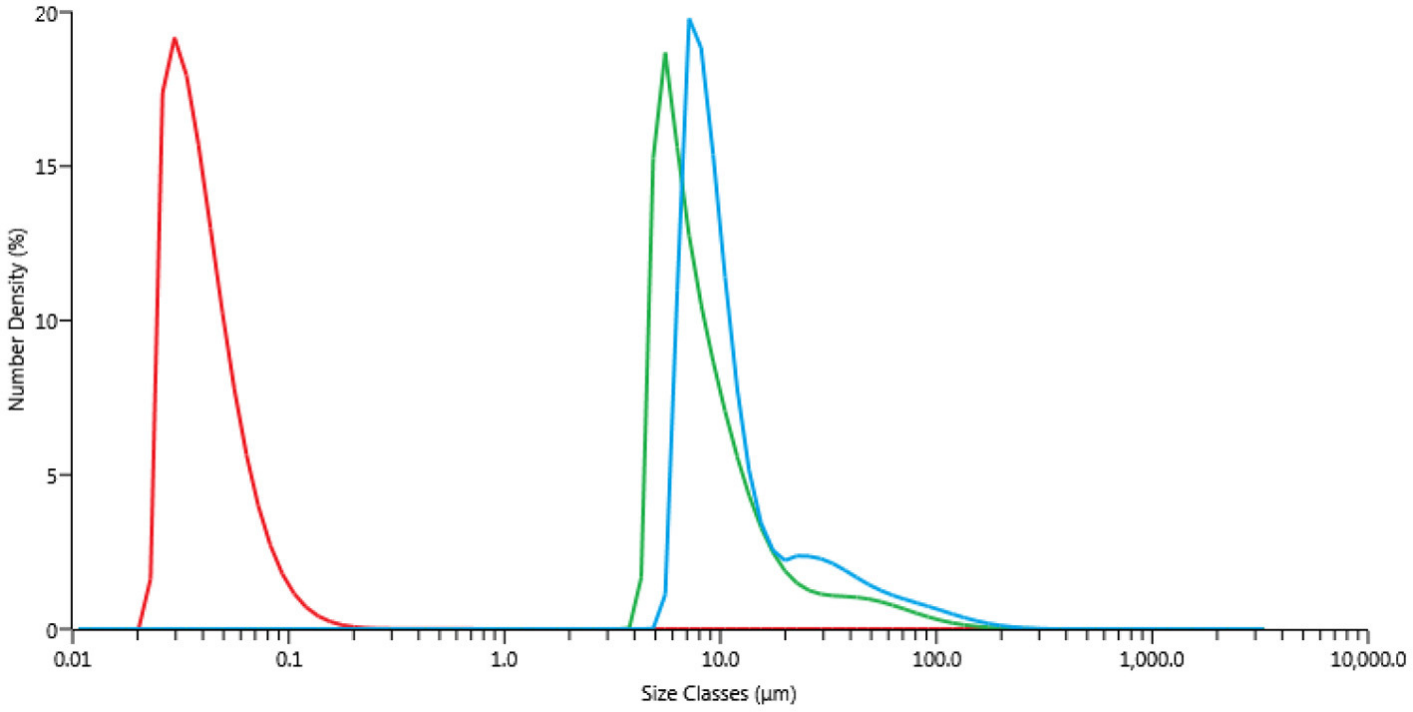
Particle-size distributions, PSD. Blue curve freshly prepared CuNPs. Green curve freshly prepared CuNPs after heating by MW irradiation for 10 min (Anton Paar Monowave 300, T = 130°C, PMax = 400 W). Red curve CuNPs after sonication for 10 min [Hielscher UP50H, F(kHz):30, P(W):50].

Freshly prepared CuNPs were sonicated for 10 min [UP50H, F(kHz):30, P(W):50] until a perfectly dispersed black solution was obtained (Supplementary Figure 8). Offline particle-size distribution measurements (based on volume) were acquired and compared with those of freshly prepared NPs (Figure 2, red and blue curves). A laser diffractometer (Malvern, MasterSizer 3000 hydro SV) was employed and particle sizes were determined by measuring the intensity of scattered light when laser beam passed through a dispersed particulate sample. 0.5 mL of the sample (*c*_NP_ = 1 g/L) was injected into 6.5 mL of deionised water in the measurement cell (so that the resulting concentration in the cell was 0.07 g/L) and mixed for 5 min using a built-in magnetic stirrer. The obtained scattering curves were averages of three subsequent measurements. Unlike the TEM observations, the Cu particles here had larger sizes due to the formation of aggregates, with an average size of 100 μm when suspended. US irradiation significantly influenced particle magnitude, with the size decreasing to 20 nm.

**Figure 3:**
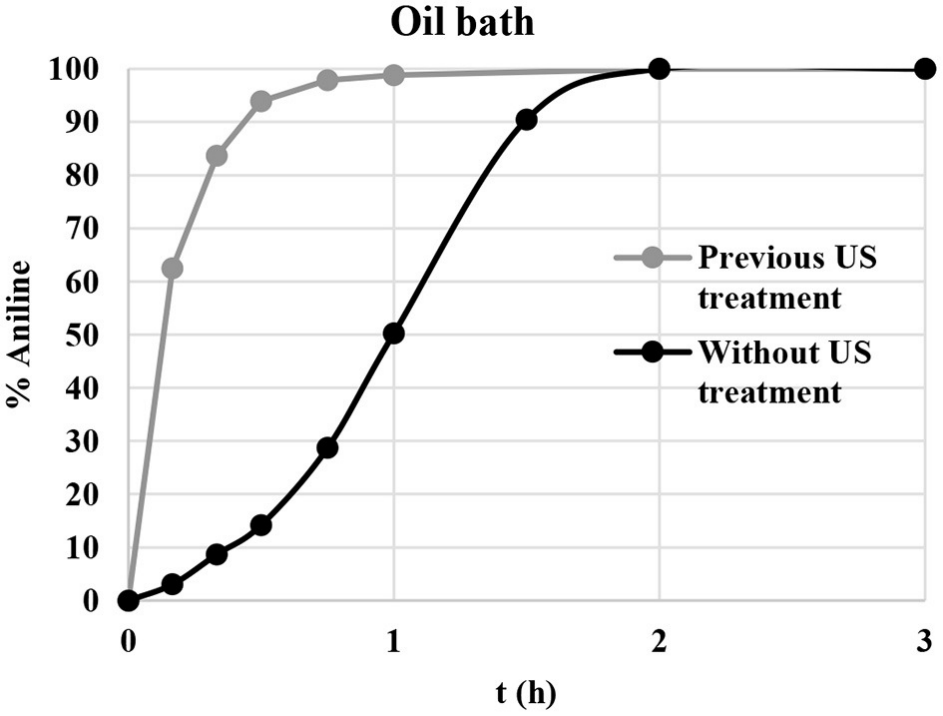
Nitrobenzene reduction profile with and without previous CuNP US-sonication. Pretreatment: CuNPs, US irradiation 10 min [Hielscher UP50H, F(kHz):30, P(W):50]. Reaction conditions: nitrobenzene (1 mmol), KOH (2 mmol), glycerol (40 mmol), CuNPs (5 mol%), 130°C. The % of aniline was determined by GC-MS.

A kinetic study was performed to assess the influence of US sonication on the reaction rate; the nitrobenzene reduction was carried out under optimal reaction conditions in the presence of freshly prepared CuNPs and pre-sonicated CuNPs. As shown in Figure 3, sigmoidal behavior was observed when the reaction was performed under conventional conditions and full conversion was obtained in 2 h. US pretreatment clearly had a significant effect on the reaction rate, and the complete conversion of nitrobenzene to aniline was achieved in 1 h. Excellent results were also obtained when the transfer hydrogenation of nitrobenzene was performed under MW irradiation, with the reaction time falling to 30 min (Table 1, entry 13). A slight effect on reaction rate was also observed when the reaction was performed under US irradiation, and full conversion was observed after 1 h (Table 1, entry 12).

### Influence of MW Irradiation

Glycerol's high boiling point combined with its low environmental impact, cost, vapor pressure and high dielectric constant make it an optimal candidate for MW-promoted organic syntheses. Furthermore, as described in (Van De Kruijs et al., 2010), the interactions between MW and the heterogeneous metal-catalyst particles generates electrostatic discharges that can lead to the formation of active particles in the reaction media (Van De Kruijs et al., 2010; Horikoshi and Serpone, 2014). It is for both of these reasons that MW irradiation was chosen for the optimization of the CuNPs-catalyzed transfer hydrogenation of nitrobenzene in glycerol.

A preliminary evaluation of the influence of MW irradiation on the nitrobenzene reduction was performed in a multimode MW oven (*CEM Mars 5, max*. power 400 W). The reaction was carried out at 130°C, and nitrobenzene was completely converted to aniline after 30 min (Table 1, entry 13).

**Figure 4:**
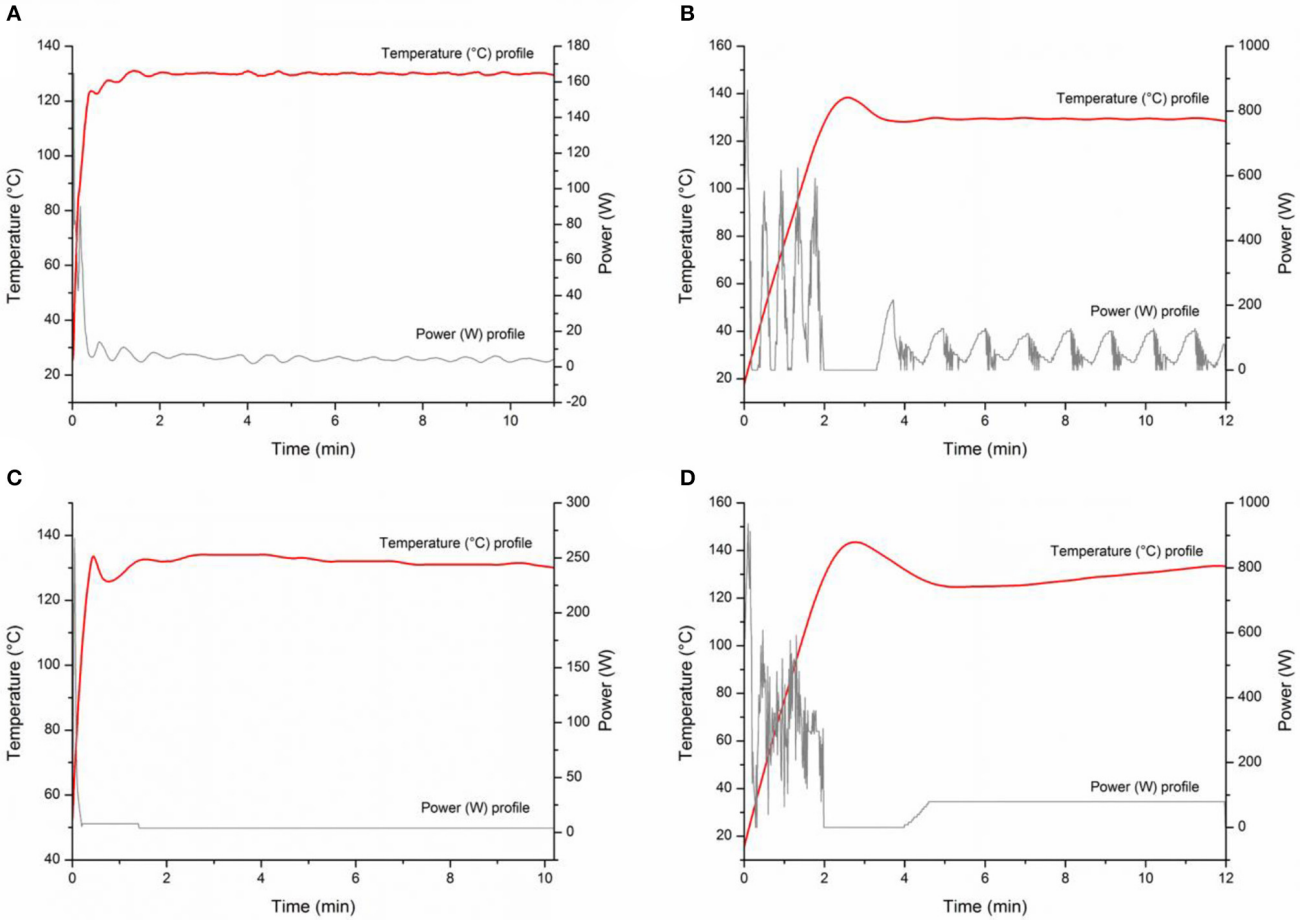
Temperature and power profile curves registered by MW instruments: **(A)** Anton Paar Monowave 300, Program: 2 min P_max_ = 100% heated as quickly as possible to reach 130°C, then T = 130°C; **(B)** Milestone MicroSynth, Program: 2 min P_max_ = 400 W heated as quickly as possible to reach 130°C, then T = 130°C; **(C)** Anton Paar Monowave 300, Program: 2 min P_max_ = 100% heated as quickly as possible to reach 130°C, then P: 4W; **(D)** Milestone MicroSynth, Program: 2 min P_max_ = 400 W heated as quickly as possible to reach 130°C, then P: 80 W.

In order to identify the best technology for reaction scale-up, a number of MW devices were used for MW-assisted batch syntheses of aniline. Reactions were performed in constant temperature mode and, separately, in constant power mode in two monomode MW instruments (Anton Paar Monowave 300 and CEM Discover SP) and two multimode MW systems (CEM Mars 5 and Milestone MicroSynth). When constant temperature mode was used, the power was automatically adjusted to reach the set temperature as quickly as possible and then maintain it using a dynamic feedback power loop. In constant power mode, the instrument adjusted the power to reach the reaction temperature as quickly as possible and then set the chosen constant power (Figure 4, gray profiles). In order to maintain the desired temperature, the selected power was carefully evaluated in advance using trial-error methodology. As demonstrated in Figure 4, the constant power set for monomode MW instruments was 4 W, while 80 W was needed in the multimode MW. As depicted in Table 2, while the reactions were complete after 30 min in all the experiments, substantial differences after 15 min of reaction time depending on the type of MW cavity (monomode vs. multimode MW cavity) and on the temporal heating profile (constant power vs. constant temperature) were observed. Better results were always obtained after 15 min when the power was maintained constant (Table 2, entries 2, 4, 6, 8). However, only in multimode-assisted reactors was full conversion obtained. The temperature and power profiles in all the MW instruments were registered when working with both methods: fixed temperature (Figures 4A,B) and fixed power (Figures 4C,D). Multimode systems have lower power density than monomode devices because of their large chambers, and higher power (80 W) is therefore required to bring the reaction mixture to the desired temperature. Higher MW power produces higher conversions, as MW electron-field effects are very important, meaning that electrostatic discharges can be generated via the interaction between MWs and the heterogeneous CuNPs, leading to the formation of active species in the reaction media (Horikoshi et al., 2011). The differences observed when the reaction time was shortened to 15 min may be due to the amount of energy provided to the sample, as demonstrated by the fluctuation in the power provided when the system is set to work at constant temperature (see Figures 4A,B), resulting in worse conversions than those obtained with constant power. As depicted in Table 2, the results of the reactions in the two monomode instruments (CEM Discover and Anton Paar Monowave 300) were similar to each other, as were those of the reactions in the two multimode instruments (CEM Mars and Milestone Microsynth). In fact, reaction outcome did not depend on the instrument used, but differences between the monomode and multimode reactors were observed.

**Table 2:** MW-assisted nitrobenzene reduction optimization.

**Entry**	**Method**	**System**	**Apparatus**	**Reaction time (min)**	**Yield (%)^a^**
1	MW-T constant^b^	Anton Paar Monowave 300	Monomode	15	21
				30	>99
2	MW-P constant^c^	Anton Paar Monowave 300	Monomode	15	49
				30	>99
3	MW-T constant^b^	CEM, Discover	Monomode	15	22
				30	>99
4	MW-P constant^c^	CEM, Discover	Monomode	15	53
				30	>99
5	MW-T constant^b^	CEM, MARS	Multimode	15	52
				30	>99
6	MW-P constant^d^	CEM, MARS	Multimode	15	>99
				30	>99
7	MW-T constant^b^	Milestone, MicroSynth	Multimode	15	58
				30	>99
8	MW-P constant^d^	Milestone, MicroSynth	Multimode	15	>99
				30	>99

*Reaction conditions: CuNPs (5 mol%) and glycerol were sonicated for 10 min, creating a perfectly dispersed black solution. Nitrobenzene (1 eq) and KOH (2 eq) where then added*.

a *Determined by GC-MS*.

b *Program: 2 min P_max_ = 400 W heated as quickly as possible to reach 130°C, then T = 130°C*.

c *Program: 2 min P_max_ = 100% heated as quickly as possible to reach 130°C, then P: 4 W*.

d *Program: 2 min P_max_ = 400 W heated as quickly as possible to reach 130°C, then P: 80 W*.

**Figure 5:**
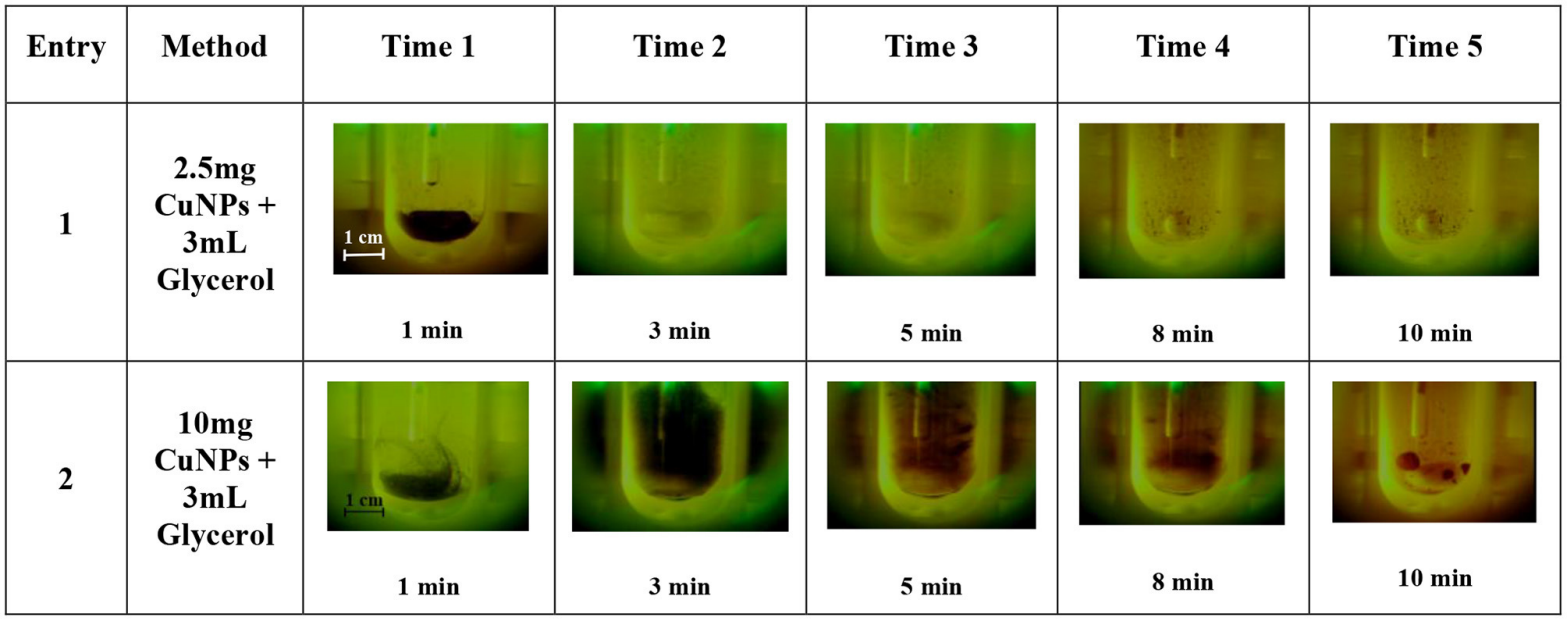
CuNPs behavior when applying MW-irradiation (Anton Paar Monowave 300, Program: 2 min Pmax = 100% heated as quickly as possible to reach 130°C, then T = 130°C). Recorded using USB Digital Microscope Supereyes B003+.

One of the main issues when working with Cu^0^-based NPs is their susceptibility to oxidation. Agglomeration is yet another effect that is usually observed when working with these small particles. Although the influence of MW irradiation on NP agglomeration has not yet been thoroughly studied, a few publications by Serpone et al., have attempted to evaluate the formation of aggregates of activated-carbon-supported Pd as caused by an excessive number of hot spots. In this work, some experiments were carried out in a MW-reactor, Anton Paar Monowave 300, that was equipped with a USB Digital Microscope Supereyes B003+ in order to better understand CuNP behavior inside the MW cavities. This multi-function microscope allowed us to follow the reaction when MW irradiation was applied. Firstly, glycerol was added to the test tube together with sequentially increasing quantities of CuNPs (2.5, 10, 20, and 40 mg). As showed in Figure 5 entries 1–2, the brown-dark CuNPs were suspended in glycerol and after 3 min were dispersed in the solvent. This behavior depends by the high viscosity at room temperature of glycerol that decrease by heating. After approximately 2 min of irradiation the magnetic bar started an efficient stirring. As can be observed, the concentration of CuNPs highly influenced aggregation, and the precipitation of large particles can be observed after a few minutes when working with 10 mg/3 mL or higher concentrations. The particle-size distribution of CuNPs was therefore measured after the MW-promoted reduction of nitrobenzene in glycerol was performed in the same instrument (Anton Paar Monowave 300), and a similar size-distribution profile to that of freshly prepared NPs was detected (Figure 2, green profile).

### MW Scale-Up

The scale-up of MW heating constitutes a growing demand for industry thanks to the successes achieved on the lab scale. There are two known paths for this task: the continuous-flow method and batchwise. At 2.45 GHz, the most commonly used MW frequency, the MW-penetration depth in common polar solvents is around a few centimeters. Because of this, the heating of bulk samples using MW irradiation has several limitations. Controlling the stirring rate to ensure and maintain the homogeneity of a solution and avoid thermal gradients must therefore be considered. As observed in the literature, most of the reactions that are accomplished under MW irradiation are performed at high temperatures in sealed vessels. Several approaches with different processing techniques can be used to scale-up a wide range of MW-promoted reactions (Bowman et al., 2008), and the use of open reaction vessels in batch mode offers operational advantages that can address scale-up needs. The small cavity of monomode MW apparatus has to be replaced with a larger multimode unit when high volumes are processed. The reduction of nitrobenzene to aniline, which has already been optimized for a 15 mL volume (1 mmol of substrate) in our study, was scaled up to 500 mL to perform the scale-up experiments.

**Figure 6:**
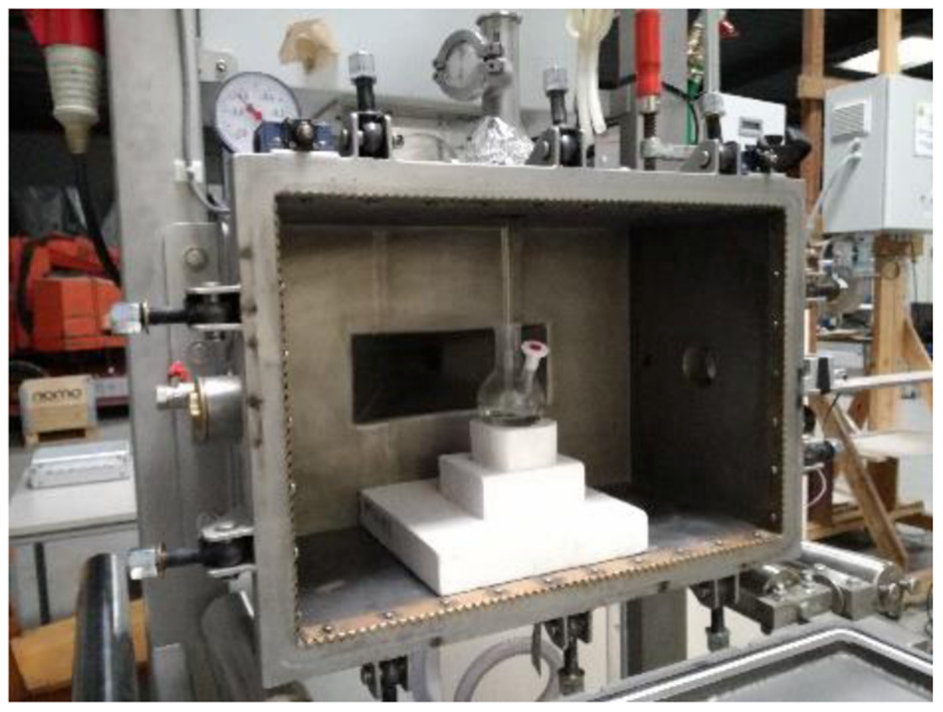
First generation multimode MW reactor. Internal cavity: 72L Power: 1.2 kW. MEAM Explorer VP.

All scale-up experiments were performed in a MW instrument MEAM Explorer VP (http://www.meam.be/; Figure 6), which is a MW multimode oven designed as a multipurpose test device for various applications. This unique design provides access on four sides of the cavity, allowing multiple connections to be used for sensors and entrances/exits for gases or liquid products. In this case, the opening on the right side was used to measure the IR temperature of the sample, while the opening on the top was used to insert the glass stirring rod. Emissivity can be defined as the effectiveness of emitting energy in form of thermal irradiation and varies from 0 to 1. It is of high significance when the temperature is measured with an IR camera. Transmissivity refers to the proportion of the radiation that hits a body and ends up being transmitted through it without being absorbed or reflected, and describes the level of infrared radiation that permeates the object. While the emissivity value is intrinsic and only depends on sample nature, the transmissivity value also depends on the shape of the vessel and its material. Both transmissivity and emissivity have to be measured to ensure the optimal calibration of the IR camera, allowing the temperature measured by the system to be as accurate as possible. The emissivity and transmissivity of the reaction mixture were determined by comparing the solution temperature measured by a thermocouple and an IR camera; the factors were 0.95 and 0.48, respectively. It is important to always place the IR camera in the same location in order to maintain constant parameter values. Once those parameters were obtained, a number of experiments were performed.

**Figure 7:**
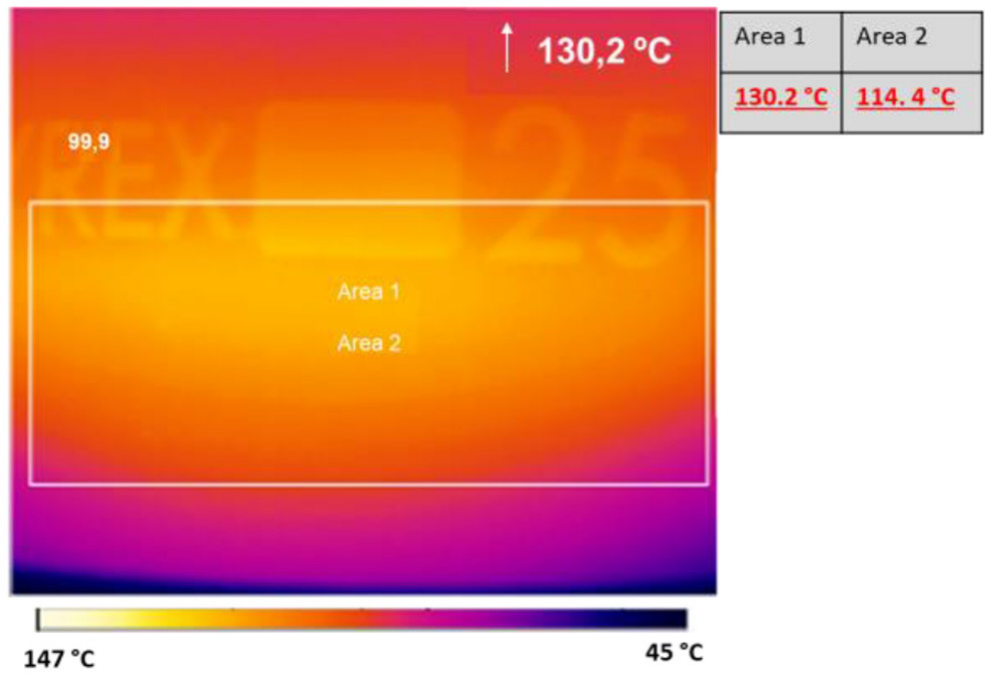
Histogram density of temperature recorded by IR camera Optris IP Connect. Reaction time: 10 min.

**Figure 8:**
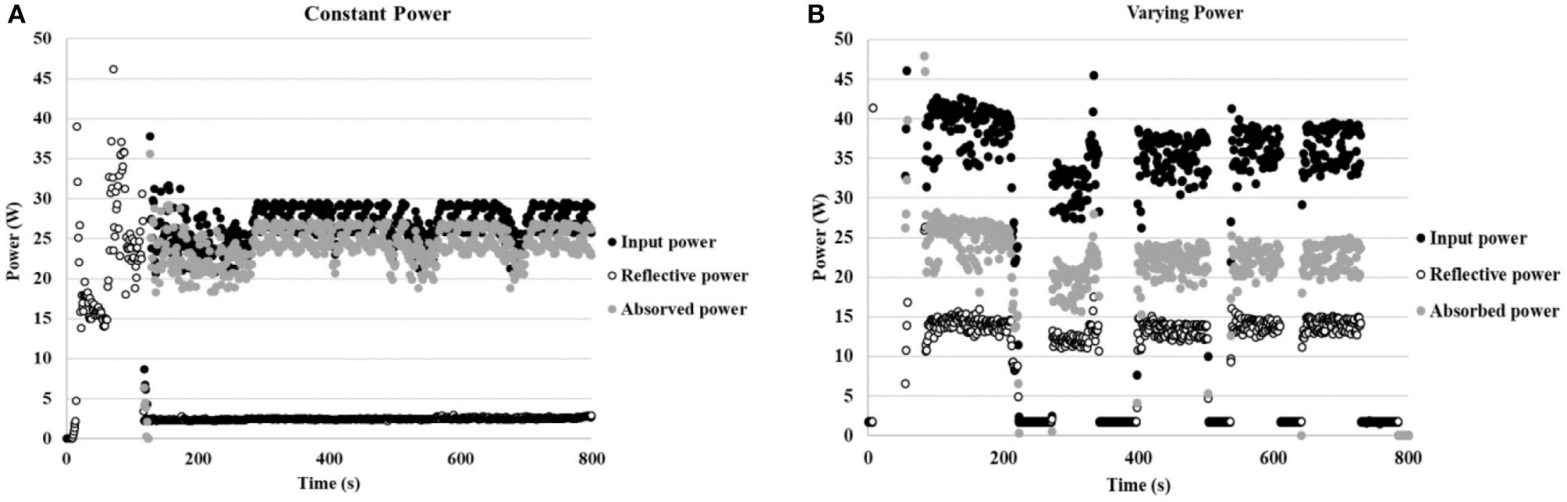
**(A)** Power profile while working at constant power (25–30 W). **(B)** Power profile while working at varying power (40–0 W) (the power meter displays 0–4 W when the magnetron is completely off).

Preliminarily, the reaction was carried out at 130°C in a 250 mL round-bottom flask and a glass stick was used to stir the 90 mL solution (6 mmol). Two reactions were performed: one experiment was carried out at constant power (25–30 W), while power was varied in the second (from 40 to 0 W). The temperature was maintained constant at 130°C in both experiments. The histogram density of temperature in the cavity was registered using an IR camera (Optris PI Connect, Figure 7). As indicated by the temperatures next to the original figure, the maximum temperature inside the flask is 130°C and the average of area 2, bulk volume inside the flask, is 114°C. The input power and reflective power were measured in order to identify the power value that was actually absorbed by the reaction solution. As depicted in Figure 8, a higher percentage of the delivered power was absorbed when power was maintained constant (Figure 8A compared to Figure 8B).

A number of reactions were performed to optimize the MW-heating protocol and scale-up the reaction. Results are summarized in Table 3. A 50% yield of aniline was obtained after 20 min of MW irradiation when working with constant power when the reaction was performed on the 6 mmol scale (90 mL glycerol) in the presence of 10 mol% of CuNPs. The conversion was reduced to 35% with varying power, which demonstrates that the efficacy of MW promotion on the Cu-catalyzed transfer hydrogenation of nitrobenzene is reduced when fluctuating power is used. In agreement with our previous results on the laboratory scale, the pretreatment of CuNPs with US significantly enhanced the reaction rate (Table 3, entry 3); 66% reduced product was achieved in this case.

**Table 3:** Nitrobenzene reduction optimization.

**Entry**	**Method**	**Scale (mmol)/mL glycerol**	**Catalyst (mol%)**	**T (°C)**	**t (min)**	**Yield (%)^a^**
1	MW (25–30 W)	6 mmol/90 mL	10	130	20	50
2	MW (40–0 W)	6 mmol/90 mL	10	130	20	35
3	Presonicated CuNPs, MW (25–30 W)^b^	6 mmol/90 mL	10	130	20	66
4	Presonicated CuNPs, MW (25–30 W)^b^	6 mmol/90 mL	5	130	20	60
5	Presonicated CuNPs, MW (25–30 W)^b^	6 mmol/90 mL	5	150	20	78
6	Presonicated CuNPs, MW (25–30 W)^b^	6 mmol/90 mL	5	150	45	>99
7	Presonicated CuNPs, MW (25–30 W)^b^	18 mmol/270 mL	5	150	45	93
8	Presonicated CuNPs, MW (25–30 W)^b^	18 mmol/270mL	5	150	60	>99
9	Presonicated CuNPs, MW (25–30 W)^b^	36 mmol/540mL	5	150	60	95

*Scale-up in an industrial multimode MW. Reaction conditions: nitrobenzene (1 eq), glycerol (200 eq), KOH (2 eq)*.

a *Determined by GC-MS*.

b *CuNPs were added into the glycerol and sonicated for 10 min, forming a perfectly dispersed black solution*.

When presonicated, the amount of catalyst could be reduced from 10 to 5 mol% without a significant decrease in yield (Table 3, entry 4), while an increase in reaction temperature to 150°C yielded 78% amino derivative in 20 min. As shown, full conversion of nitrobenzene to aniline was obtained when the reaction time was increased to 45 min (Table 3, entry 6). When the reaction was performed on a larger scale (18 mmol nitrobenzene/270 mL of glycerol), the reaction time was increased to 1 h in order to complete the reduction. Moreover, the reaction was also performed in a 1 L flask (36 mmol/540 mL of glycerol) with the initial solution being sonicated. A 95% yield was achieved once this reaction mixture was heated for a total time of 1 h.

## Conclusions

Glycerol has been studied as a hydrogen donor for the exhaustive, fast and reproducible Cu-catalyzed transfer hydrogenation of nitrobenzene to aniline. Small size, roundish-shape CuNPs were prepared in glycerol and, using HRTEM, it was possible to observe that the polyol layer mediates the interaction between the metal active sites and stabilizes NP function. NP dispersion in glycerol was promoted by US irradiation and excellent results (complete conversion and >95% yield) were obtained after 2 h when CuNPs were employed for nitrobenzene reduction under conventional heating conditions at 130°C. The high polarity and low vapor pressure of glycerol allowed the effects of MW irradiation to be fully explored and, gratifyingly, the reaction was shortened to 15 min. On the basis of this detailed study, a constant power MW protocol has been optimized and the reaction was scaled-up to 36 mmol/500 mL of glycerol in a multimode industrial MW reactor.

## Materials and Methods

### General

All commercially available reagents and solvents were used without further purification. Reactions were monitored by TLC on Merck 60 F254 (0.25 mm) plates (Milan, Italy), which were visualized by UV inspection and/or by heating after spraying with 0.5% ninhydrin in ethanol. Reactions were carried out in a conventional oil bath by magnetic stirring, under US irradiation (Hielscher *Ultrasonic horn UP50H*) (Supplementary Figure 1) and in several MW devices, which operated in both monomode (Anton Paar 300, CEM Discover SP) (Supplementary Figures 2, 3), and multimode (CEM Mars 5 and MicroSynth) (Supplementary Figures 4, 5). A 1.2kW Multimode MW oven was used (Supplementary Figure 6), was used for MW-assisted reaction scale-up. NMR spectra (300 MHz and 75 MHz for ^1^H and ^13^C, respectively) were recorded. Chemical shifts were calibrated to the residual proton and carbon resonances of the solvent, CDCl_3_ (δH = 7.26, δC = 77.16). Chemical shifts (δ) are given in ppm, and coupling constants (J) in Hz. GC-MS analyses were performed in a GC Agilent 6890 (Agilent Technologies, Santa Clara, CA, USA), which was fitted with a mass detector Agilent Network 5973, using a 30 m capillary column, i.d. of 0.25 mm and film thickness 0.25 μm. GC conditions were: injection split 1:10, injector temperature 250°C, detector temperature 280°C. Gas carrier: helium (1.2 mL/min). Temperature program: from 50°C (5 min) to 100°C (1 min) at 10°C/min, to 230°C (1 min) at 20°C/min, to 300°C (5 min) at 20°C/min. HRMS was determined using MALDI-TOF mass spectra (Bruker Ultraflex TOF mass spectrometer, Milan, Italy).

Copper nanoparticles were characterized by transmission electron microscopy (TEM) and high resolution TEM (HR-TEM). The measurements were carried out using a JEOL 3010-UHR instrument operating at 300 kV and equipped with a LaB_6_ filament. Digital micrographs were acquired using a Gatan (2k × 2k)-pixel Ultrascan1000 CCD camera and were processed using Gatan digital micrograph. In order to obtain good sample dispersion and avoid modifications that may be induced by the use of a solvent, the powders were briefly contacted with the Cu grids, which were coated with lacey carbon, resulting in some particles adhering to the grid via electrostatic interactions.

### General Procedure for the Synthesis of Copper Nanoparticles

A Copper (II) sulfate solution (1.5 mL of a 0.01 M solution in water/glycerol 5:1) was stirred and an aqueous 2 M NaOH solution was added dropwise to adjust the solution pH up to 11. After stirring for 10 min, 0.5 M NaBH_4_ in water was added. Initially, the deep blue solution gradually became colorless to then turn burgundy, which indicates the formation of the copper colloid. The CuNPs were filtered on a Büchner funnel with a sintered glass disc using water and methanol to wash the catalyst.

### Nitrobenzene Reduction to Aniline

#### Optimized Nitrobenzene-Reduction Procedure Under Conventional Heating

CuNPs (3 mg, 5 mol%) were sonicated in a rounded-bottom flask with 3 mL of glycerol for 10 min [Hielscher *Ultrasonic horn UP50H, F(kHz):30, P(W):50*]. A perfectly dispersed black solution was observed. KOH (112 mg, 2 mmol) and nitrobenzene (123 mg, 1 mmol) were then added and the reaction was heated at 130°C under magnetic stirring for 2 h. The reaction mixture was cooled to room temperature and filtered to remove CuNPs. Ten milliliters of water was added and extracted with ethyl acetate (2 × 10 mL). Aqueous HCl (0.01 M) was added to the organic phase and, after extraction, the aqueous phase was basified with NaOH (0.01 M), extracted with ethyl acetate (3 × 20 mL) and dried (Na_2_SO_4_). The product was analyzed using ^1^H NMR and ^13^C NMR spectroscopy. Isolated yield 97% (Supplementary Figures 9, 10).

#### General Procedure for US-Assisted Nitrobenzene Reduction

Nitrobenzene (625 mg, 5 mmol), KOH (560 mg, 10 mmol), and the nano-copper catalyst (15 mg, 5 mol%) were added to 15 mL of glycerol and the mixture was sonicated using a Hielscher *Ultrasonic horn UP50H*, F(kHz):30, P(W):50 for 1 h. The reaction mixture was cooled down to room temperature and filtered to remove the CuNPs. Thirty milliliters of water was added and extracted with ethyl acetate (2 × 30 mL). Aqueous HCl (30 mL, 0.01 M) was added to the organic phase and, after extraction, the aqueous phase was basified with NaOH (0.01 M), extracted with ethyl acetate (3 × 60 mL) and dried (Na_2_SO_4_). The product was analyzed using ^1^H NMR and ^13^C NMR spectroscopy. Isolated yield 98% (Supplementary Figures 9, 10).

#### General Procedure for MW-Assisted Nitrobenzene Reduction

When prior catalyst dispersion was required, the CuNPs (15 mg, 5 mol%) were weighed, added to a rounded-bottom flask with 15 mL of glycerol and sonicated for 10 min [Hielscher *Ultrasonic horn UP50H, F(kHz):30, P(W):50*]. A perfectly dispersed black solution was observed. KOH (560 mg, 10 mmol) and nitrobenzene (625 mg, 5 mmol) were then added and the reaction was carried out. Homogeneous MW distribution was ensured by a magnetic stirrer. Several MW devices were employed; both monomode systems (Anton Paar Monowave 300 and CEM Discover SP) and multimode systems (CEM Mars 5 and Milestone MicroSynth). Two different methods were used to apply MW irradiation: (a) fixed temperature and (b) fixed power.

- Monomode systems (Anton Paar Monowave 300, CEM Discover SP): 2 min were required to reach the reaction temperature (130°C) using the program: “heat as quickly as possible” (Maximum power 400 W). When performing the reaction at fixed temperature, the program “hold” was selected in order to main the temperature constant (130°C) during the reaction. In this mode, the MW-reactor automatically adjusts the power to reach the indicated temperature. Reaction time: 30 min. When performing the reaction at fixed power, the program “constant power” was selected (4 W). In this way, a constant set power maintains the reaction mixture at the desired temperature (130°C). Reaction time: 15 min.
- Multimode systems (CEM Mars 5 and Milestone MicroSynth): 2 min were required to reach the reaction temperature (130°C) (Maximum power 400 W) (CEM Mars 5: Power 100%). When performing the reaction at fixed temperature, the power was set at 400 W, and 130°C was selected as the constant temperature. The MW-reactor could then automatically adjust the power. Reaction time: 30 min. When the reaction was performed at fixed power, the mixture was irradiated at constant power (80 W) for the whole reaction time. Reaction time: 15 min.

The reaction mixture was cooled to room temperature and filtered to remove the CuNPs. Thirty milliliters of water were added and extracted with ethyl acetate (2 × 30 mL). Aqueous HCl (30 mL, 0.01 M) was added to the organic phase, and, after extraction, the aqueous phase was basified with NaOH (0.01 M), extracted with ethyl acetate (3 × 60 mL) and dried (Na_2_SO_4_). The product was analyzed using ^1^H NMR and ^13^C NMR spectroscopy. The isolated yield was 98%. When using the Anton Paar 300 reactor, the reaction temperature was controlled simultaneously by a ruby thermometer (a fiber optic sensor that is immersed in the reaction mixture and accurately measures the internal temperature over the entire reaction process) and an IR sensor that provides a measurement of the external temperature of the reaction vials. When using the CEM Discover SP, the temperature was measured by an IR sensor, and fiber optics where installed in both multimode systems (CEM Mars 5 and MicroSynth).

#### General Procedure for MW-Assisted Nitrobenzene Reduction. Scale-Up

Scale up experiments were performed in the MW multimode instrument MEAM Explorer VP (1.2 kW). The emissivity (0.95) and transmissivity (0.48) of the reaction solvent (glycerol) were determined, first of all, to ensure the optimal calibration of the IR camera and, therefore, ensure the measured reaction temperature. These values remained constant when measured with the reaction mixture. The IR camera was always set in the same position. CuNPs (18 mg, 5 mol%) were sonicated in a rounded-bottom flask with glycerol (90 mL) for 10 min [Hielscher *Ultrasonic horn UP50H, F(kHz):30, P(W):50*]. A perfectly dispersed black solution was observed. KOH (672 mg, 12 mmol) and nitrobenzene (748 mg, 6 mmol) were then added and the reaction was carried out. Suitable reagent quantities were added when the reaction was scaled-up to 18 and 36 mmol of nitrobenzene: glycerol (200 eq), KOH (2 eq) and CuNPs (5 mol%). Homogeneous MW distribution was ensured by a stirring bar. When performing the reaction at fixed temperature, 2 min (P_max_ = 100%) were required to achieve the reaction temperature (130°C). In this mode, the MW-reactor automatically adjusted the power (40–0 W) to maintain the desired temperature (130°C) for the whole reaction. When the reaction was performed at fixed power, 25–30 W was maintained. In this way, a constant set power maintained the reaction mixture at the desired temperature (130°C). Reaction time: 45 min (6 mmol Nitrobenzene), 60 min (18 mmol and 36 mmol). Reaction workup was performed as described in the previous MW-promoted procedure.

## Data Availability Statement

All datasets generated for this study are included in the article/Supplementary Material.

## Author Contributions

KM, GC, and GS: methodology and experimental design. MJM, JJ, and VG: investigation. KM, VG, and CG: data curation. MM: catalyst analysis. MJM and KM: writing—original draft preparation. GC, TG, GS: writing—review and editing.

### Conflict of Interest

VG and CG were employed by the company MEAM Microwave Test Center, Industrieweg 1119, 3540 Herk-de-Stad, Belgium. The remaining authors declare that the research was conducted in the absence of any commercial or financial relationships that could be construed as a potential conflict of interest.
