# Supplementary material for: Glycerol: An Optimal Hydrogen Source for Microwave-Promoted Cu-Catalyzed Transfer Hydrogenation of Nitrobenzene to Aniline
Source: Front Chem. 2020 Jan 29;8:34. doi: 10.3389/fchem.2020.00034 (PMC7000456; doi:10.3389/fchem.2020.00034)
Supplement: Supplementary file 1 [file Table_1.docx]

Glycerol: an optimal hydrogen source for microwave-promoted Cu-catalysed transfer hydrogenation of nitrobenzene to aniline

Supplementary Material

Maria Jesus Moran,^1^ Katia Martina,^1^ Georgios D. Stefanidis,^2^ Jeroen Jordens,^2^ Tom Van Gerven,^2^ Vincent Goovaerts,^3^ Maela Manzoli,^1^ Carlo Grofﬁls,^3^ Giancarlo Cravotto *^1^

^1^ Dipartimento di Scienza e Tecnologia del Farmaco, University of Turin, Via Pietro Giuria 9, 10125 Turin, Italy

^2^ Department of Chemical Engineering KU Leuven, Celestijnenlaan 200F, 3001 Leuven, Belgium.

^3^ MEAM Microwave Test Center, Industrieweg 1119, 3540 Herk-de-Stad, Belgium.

*** Correspondence:**Giancarlo Cravotto
[giancarlo.cravotto@unito.it](mailto:giancarlo.cravotto@unito.it)

Index

[1 General information 2](#_Toc16323634)

[2 General procedures 4](#_Toc16323635)

[2.1 General Procedure for synthesis of copper nanoparticles 4](#_Toc16323636)

[2.2 Catalyst dispersion 5](#_Toc16323637)

[2.3 Particles size distribution. 5](#_Toc16323638)

[2.4 Nitrobenzene reduction to aniline. 6](#_Toc16323639)

[2.4.1 Optimized procedure of nitrobenzene reduction under conventional heating. 6](#_Toc16323640)

[2.4.2 Optimized procedure of Ultrasound assisted nitrobenzene reduction. 6](#_Toc16323641)

[2.4.3 Optimized procedure of Microwave assisted nitrobenzene reduction. 6](#_Toc16323642)

[2.4.4 General procedure of Microwave assisted nitrobenzene reduction. Scaling up. 7](#_Toc16323643)

[3 NMR Spectra: 7](#_Toc16323644)

# General information

All commercially available reagents and solvents were used without further purification. Reactions were monitored by TLC on Merck 60 F254 (0.25 mm) plates (Milan, Italy), which were visualized by UV inspection and/or by heating after a spraying with 0.5% ninhydrin in ethanol. Reactions were carried out in conventional oil bath by magnetic stirring, under US irradiation (Hielscher Ultrasonic horn UP50H) (Figure 1) and under different MW devices, both monomode (Anton Paar Monowave 300, CEM Discover SP) (Figure 2 and 3) and multimode (CEM Mars 5 and Milestone MicroSynth) (Figure 4 and 5). For MW-assisted reaction scale up a 1.2kW Multimode MW was used located in the MEAM Test and Training Center, based in Herk-de-Stad in Belgium (Figure 6). NMR spectra (300 MHz and 75 MHz for ^1^H and ^13^C, respectively) were recorded. Chemical shifts were calibrated to the residual proton and carbon resonances of the solvent, CDCl_3_ (δH = 7.26, δC = 77.16). Chemical shifts (δ) are given in ppm, and coupling constants (J) in Hz. GC-MS analyses were performed in a GC Agilent 6890 (Agilent Technologies, Santa Clara, CA, USA) that was fitted with a mass detector Agilent Network 5973, using a 30 m capillary column, i.d. of 0.25 mm and film thickness 0.25 μm. GC conditions were: injection split 1:10, injector temperature 250 °C, detector temperature 280 °C. Gas carrier: helium (1.2 mL/min), temperature program: from 50 °C (5 min) to 100 °C (1 min) at 10 °C/min, to 230 °C (1 min) at 20 °C/min, to 300 °C (5 min) at 20 °C/min. HRMS was determined using a MALDI-TOF mass spectra (Bruker Ultraflex TOF mass spectrometer, Milan, Italy.


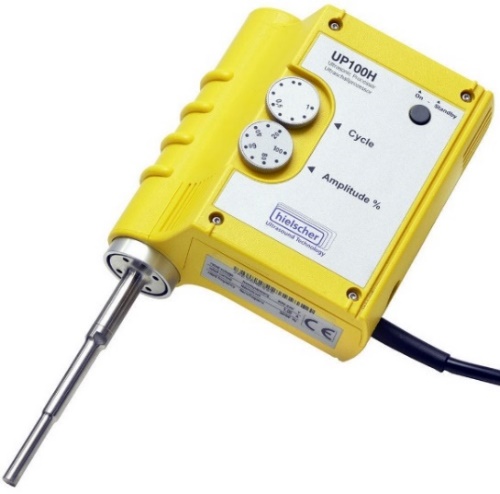
*Figure 1: Hielscher* Ultrasonic horn UP50Hz


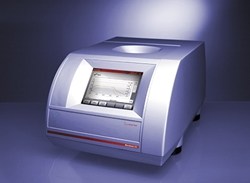

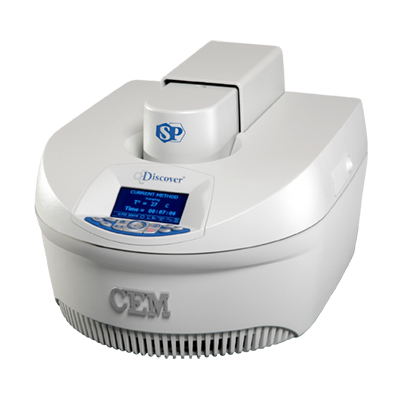


*Figure 2 and 3: Anton Paar Monowave 300 and CEM Discover SP*


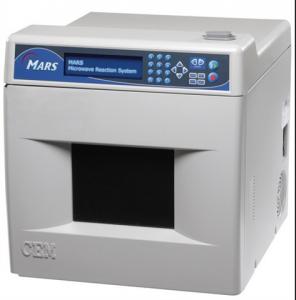

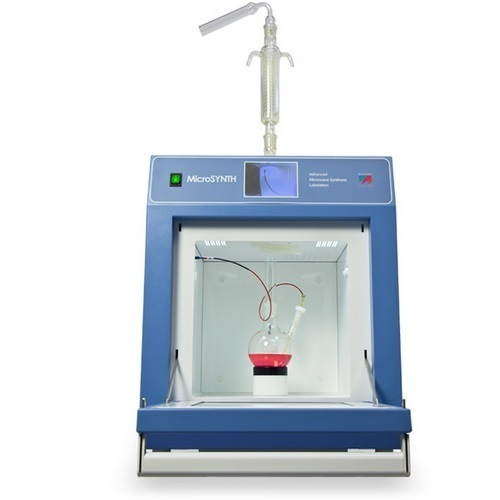


*Figure 4 and 5: CEM Mars 5 and Milestone MicroSynth*


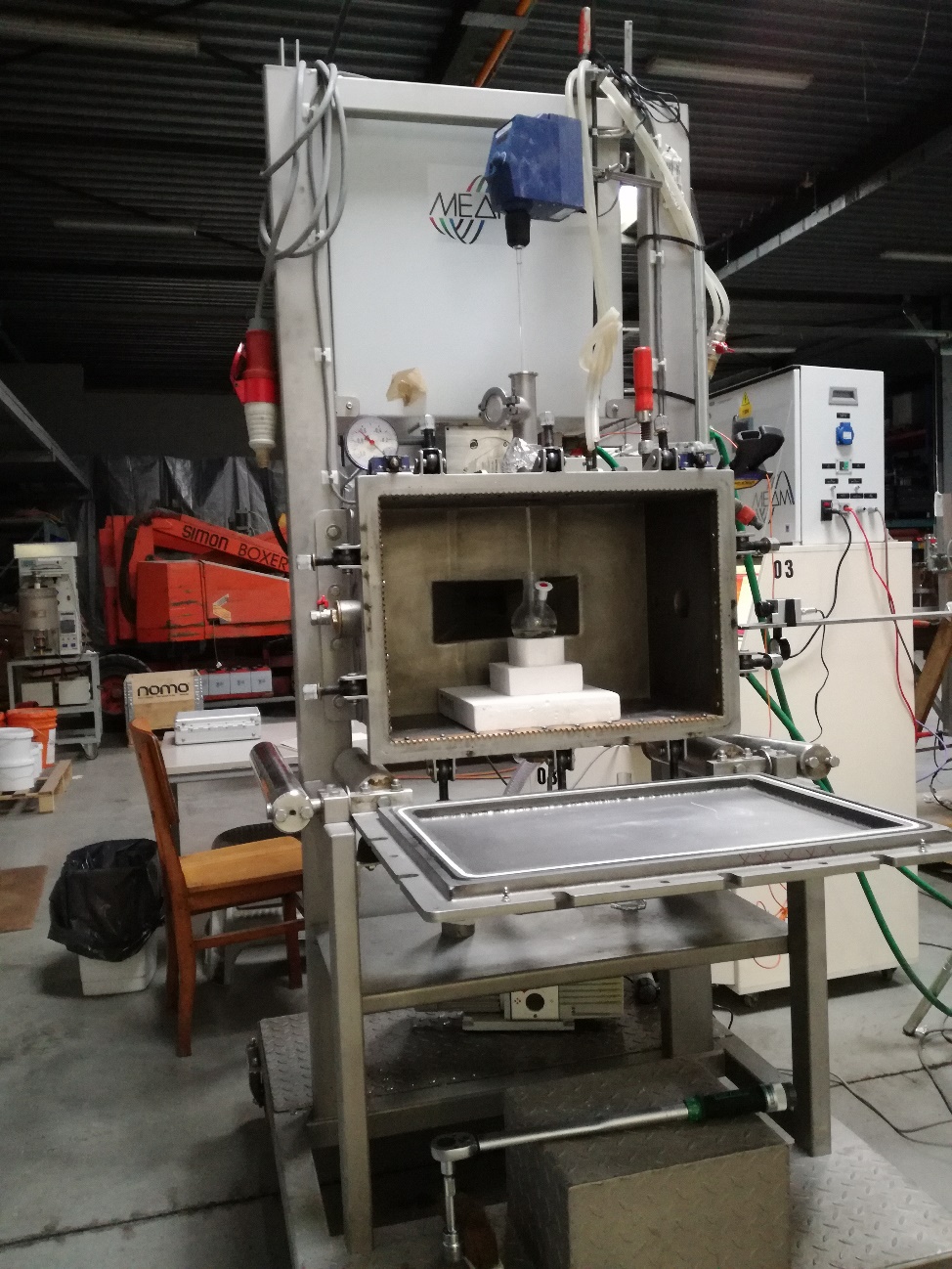


*Figure 6: MW-reactor for scaling up.*

# General procedures

## General Procedure for synthesis of copper nanoparticles

A Copper (II) sulfate solution (1.5 mL of a 0.01 M solution in water/glycerol 5:1) was stirred and was followed dropwise by addition of 2 M NaOH aqueous solution to adjust the solution pH up to 11. After stirring for 10 min, 0.5 M NaBH_4_ in water was added into the flask. Firstly, the deep blue solution gradually became colourless, and then it turned burgundy, which shows the formation of copper colloid. The copper nanoparticles were filtered on a Büchner funnel with a sintered glass disc using water and methanol to wash the catalyst.

Copper nanoparticles were characterized by transmission electron microscopy (TEM) and high resolution TEM (HR-TEM). The measurements were carried out with a JEOL 3010-UHR instrument operating at 300 kV and equipped with a LaB_6_ filament. Digital micrographs were acquired by a Gatan (2k x 2k)-pixel Ultrascan1000 CCD camera and processed by Gatan digital micrograph. To obtain a good dispersion of the sample and to avoid any modification induced by the use of a solvent, the powders were briefly contacted with the Cu grids coated with lacey carbon, resulting in the adhesion of some particles to the grid by electrostatic interactions.


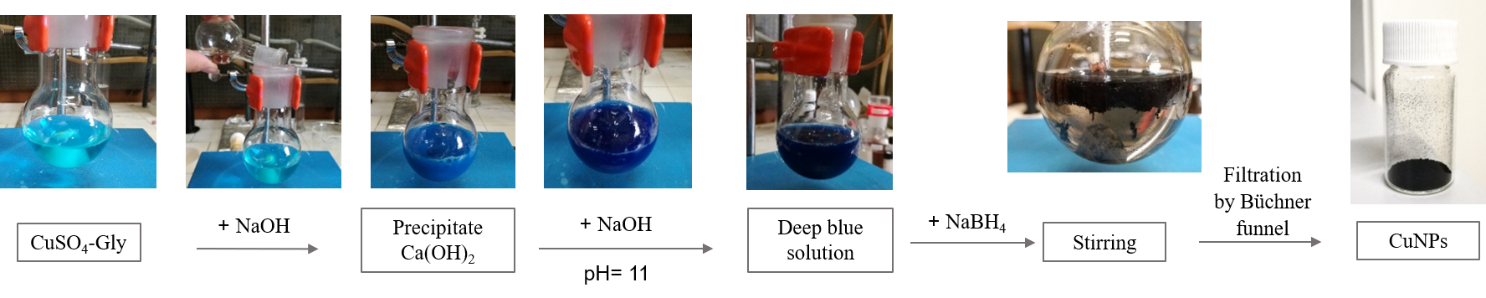

Figure 7. Figure 1: (a) HRTEM image of CuNPs, (b) zoom on a region showed in a, and (c) schematic representation of the nanoparticle morphology and graph reporting the measurement of the spacing among the observed diffraction fringes. Instrumental magnification 300000 ×.

## Catalyst dispersion

When previous catalyst dispersion was required, CuNPs (15 mg, 5 mol%) were weighted, added to a rounded-bottom flask with 15 mL of glycerol and sonicated for 10 min (*Hielscher* Ultrasonic horn UP50H, frequency 30 kHz, power 50 W). A perfectly dispersed black solution was observed. (Fig. 8).


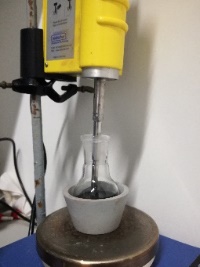

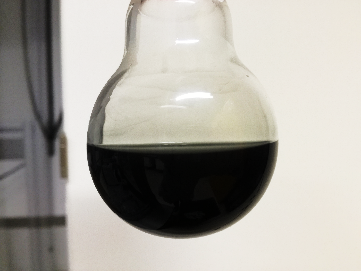


*Figure 8: CuNPs dispersion by 10 min sonication with ultrasonic horn.*

## Particles size distribution.

Freshly prepared CuNPs were sonicated for 10 min (UP50H, F(kHz):30, P(W):50) till a perfectly dispersed black solution was observed. Particle size distribution offline measurements (based on volume) were performed and compared with freshly prepared NPs (Figure 9, Curves red and blue). Particle size distribution was also measured on CuNPs after a MW promoted reaction of reduction of nitrobenzene in glycerol (Anton Paar Monowave 300) and we could observe that a similar profile of size distribution was detected if compared to freshly prepared NPs (Figure 9, Curve green). A Laser diffractometer (Malvern, MasterSizer 3000 hydro SV) was employed and the particles size was determined by measuring the intensity of light scattered as a laser beam passes through a dispersed particulate sample. 0.5 mL of the sample (*c*_NP_=1g/L) was injected in 6.5 mL of deionized water in the measurement cell (so that the resulting concentration in the cell was 0.07 g/L) and mixed for 5 min using a built-in magnetic stirrer. The obtained scattering curves were averages of three subsequent measurements.

## Nitrobenzene reduction to aniline.

***Scheme 1.*** *General scheme for the reduction of nitrobenzene to aniline.*

### Optimized procedure of nitrobenzene reduction under conventional heating.

CuNPs (3 mg, 5 mol%) in a rounded-bottom flask with 3 mL of glycerol were sonicated for 10 min (Hielscher Ultrasonic horn UP50H, F(kHz):30, P(W):50). A perfectly dispersed black solution was observed. The KOH (112 mg, 2 mmol) and the nitrobenzene (123 mg, 1 mmol) were then added and the reaction was heated at 130 °C under magnetic stirring for 2 h. The reaction mixture was cooled down to room temperature and filtered to remove CuNPs. 10 mL of water was added and extracted with ethyl acetate (2x10 mL). To the organic phase, aqueous HCl (0.01M) was added and after extraction the aqueous phase was basified with NaOH (0.01 M), extracted with ethyl acetate (3x20 mL) and dried (Na_2_SO_4_). Product was analyzed using ^1^H NMR and ^13^C NMR spectroscopy. Isolated yield 97%.

### Optimized procedure of Ultrasound assisted nitrobenzene reduction.

Nitrobenzene (625 mg, 5 mmol), KOH (560 mg, 10 mmol) and nano-copper catalyst (15 mg, 5 mol%) were added into 15 mL of glycerol and the mixture was sonicated in a Hielscher Ultrasonic horn UP50H, F(kHz):30, P(W):50 for 1 h. The reaction mixture was cooled down to room temperature and filtered to remove CuNPs. 30 mL of water was added and extracted with ethyl acetate (2x30 mL). To the organic phase, aqueous HCl (30 mL, 0.01M) was added and after extraction the aqueous phase was basified with NaOH (0.01 M), extracted with ethyl acetate (3x60 mL) and dried (Na_2_SO_4_). Product was analyzed using ^1^H NMR and ^13^C NMR spectroscopy. Isolated yield 98%.

### Optimized procedure of Microwave assisted nitrobenzene reduction.

When previous catalyst dispersion was required, CuNPs (15 mg, 5 mol%) were weighted, added to a rounded-bottom flask with 15 mL of glycerol and sonicated for 10 min (Hielscher Ultrasonic horn UP50H, F(kHz):30, P(W):50). A perfectly dispersed black solution was observed. The KOH (560 mg, 10 mmol) and the nitrobenzene (625 mg, 5 mmol) were then added and the reaction was carried out. Homogeneous MW distribution was ensured by inserting a magnetic stirrer. Different MW devices were employed: both monomode systems (Anton Paar Monowave 300 and CEM Discover SP) and multimode systems (CEM Mars 5 and Milestone MicroSynth). Two different methods were used for applying MW irradiation: a) fixed temperature and b) fixed power.

- Monomode systems (Anton Paar Monowave 300, CEM Discover SP): 2 min were required to achieve the reaction temperature (130 ºC) using the program “Heat as fast as possible” (Maximum power 400 W). When performing the reaction at fixed temperature, the program “hold” was selected in order to main the temperature constant (130 ºC) during the reaction time. In this mode the MW-reactor automatically adjusts the power to reach the indicated temperature. Reaction time: 30 min. When performing the reaction at fixed power, the program “constant power” was selected (4 W). In this way, a constant set power maintains the reaction mixture at the desired temperature (130 ºC). Reaction time: 15 min.
- Multimode systems (CEM Mars 5 and Milestone MicroSynth): 2 min were required to achieve the reaction temperature (130 ºC) (Maximum power 400 W) (CEM Mars 5: Power 100%). When performing the reaction at fixed temperature, the power was left at 400 W and 130 ºC were selected as constant temperature, so the MW-reactor could automatically adjusts the power. Reaction time: 30 min. When performing the reaction at fixed power, the reaction mixture was irradiated with constant power (80 W) for the whole reaction time. Reaction time: 15 min.

The reaction mixture was cooled down to room temperature and filtered to remove CuNPs. 30 mL of water were added and extracted with ethyl acetate (2x30 mL). To the organic phase, aqueous HCl (30 mL, 0.01M) was added and after extraction the aqueous phase was basified with NaOH (0.01 M), extracted with ethyl acetate (3x60 mL) and dried (Na_2_SO_4_). Product was analyzed using ^1^H NMR and ^13^C NMR spectroscopy. Isolated yield 98%. When using the Anton Paar Monowave 300 reactor, the reaction temperature is controlled simultaneously by a ruby thermometer (a fiber optic sensor immersed into the reaction mixture that accurately delivers the internal temperature during the whole reaction process) and the IR sensor that provides an external temperature measurement of reaction vials. When employing the CEM Discover SP, the temperature measurements were determinate by an IR sensor and when using both multimode systems (CEM Mars 5 and MicroSynth) fiber optics where installed.

### General procedure of Microwave assisted nitrobenzene reduction. Scaling up.

Scale up experiments were performed with the MW multimode instrument MEAM Explorer VP (1.2 kW). Emissivity (0.95) and transmissivity (0.48) of the reaction solvent (glycerol) were firstly determined in order to ensure an optimal calibration of the IR camera and so, to ensure the measured reaction temperature. These values remain constant when measured with the reaction mixture. The IR camera was always set in the same position. CuNPs (18 mg, 5 mol%), in a rounded-bottom flask with glycerol (90 mL) were sonicated for 10 min (Hielscher *Ultrasonic horn UP50H, F(kHz):30, P(W):50)*. A perfectly dispersed black solution was observed. The KOH (672 mg, 12 mmol) and the nitrobenzene (748 mg, 6 mmol) were then added and the reaction was carried out. When the reaction was scaled-up to 18 and 36 mmol of nitrobenzene, the pertinent reagents quantities were added: glycerol (200 eq), KOH (2 eq) and CuNPs (5 mol%). Homogeneous MW distribution was ensured by inserting a stir bar. When performing the reaction at fixed temperature, 2 min (P_max_=100%) were required to achieve the reaction temperature (130 ºC). In this mode the MW-reactor automatically adjusts the power (40 W – 0 W) to maintain constant the desired temperature (130 ºC) during the whole reaction time. When performing the reaction at fixed power, 25-30 W was maintained. In this way, a constant set power maintains the reaction mixture at the desired temperature (130 ºC). Reaction time: 45 min (6mmol Nitrobenzene), 60 min (18 mmol and 36 mmol). Reaction workup was performed as described in the previous MW promoted procedure.

# NMR Spectra:

**Aniline:** ^1^H NMR (300 MHz, CDCl_3_) δ 7.26 (2H, t, J = 6 Hz), 6.86 (1H, t, J = 9 Hz), 6.76 (2H, t, J = 6 Hz), 3.63 (2H, s) ppm; ^13^C NMR (75 MHz, CDCl_3_) δ 146.57, 129.30, 118.40, 115.12 ppm.


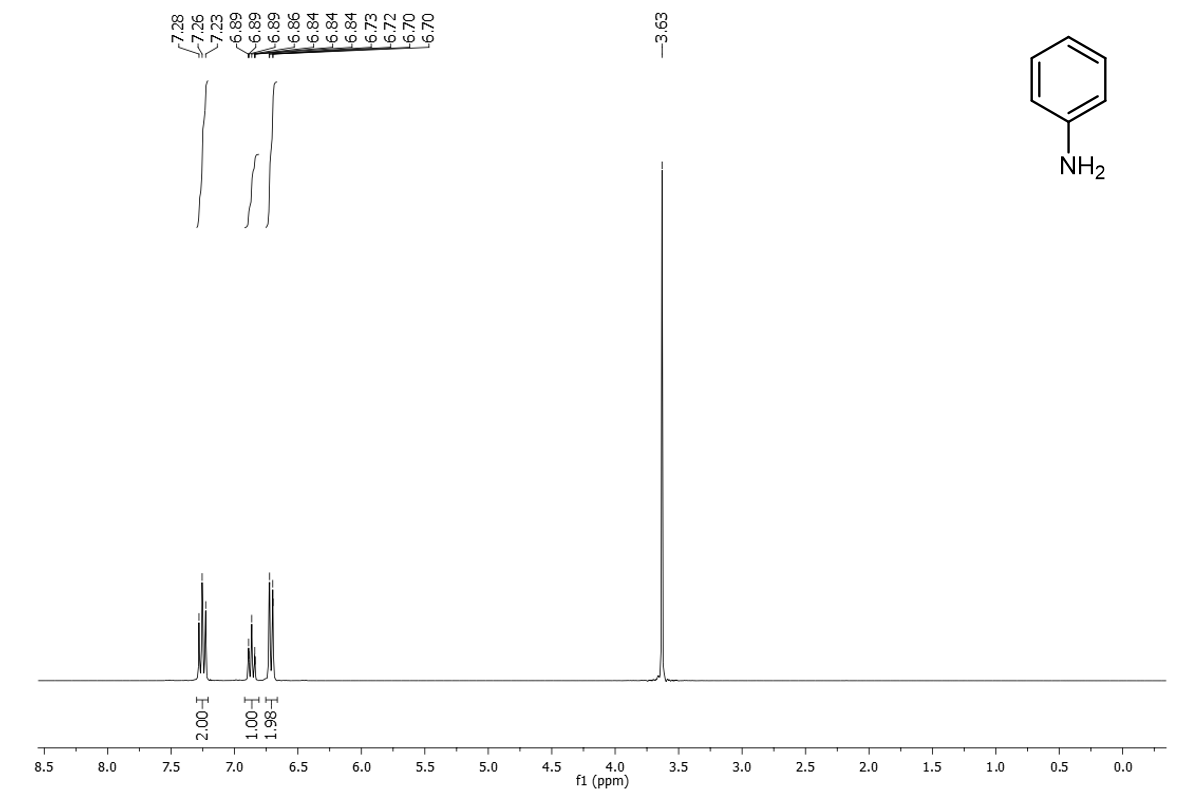


*Figure 9. ^1^H NMR (300 MHz, CDCl_3_) of Aniline*


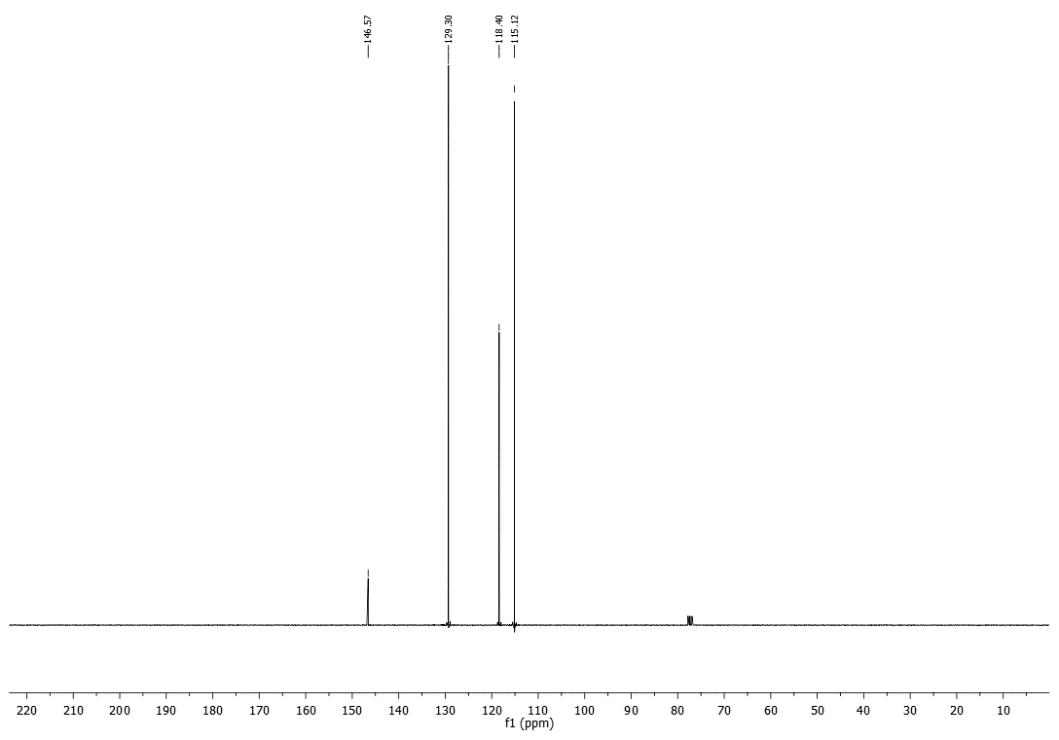


*Figure 10. ^13^C-NMR (75 MHz, CDCl_3_) of Aniline*
